# Behavioral and Metabolome Differences between C57BL/6 and DBA/2 Mouse Strains: Implications for Their Use as Models for Depression- and Anxiety-Like Phenotypes

**DOI:** 10.3390/metabo11020128

**Published:** 2021-02-23

**Authors:** Michaela D. Filiou, Markus Nussbaumer, Larysa Teplytska, Christoph W. Turck

**Affiliations:** 1 Proteomics and Biomarkers, Max Planck Institute of Psychiatry, 2-10, 80804 Munich, Germany; nussbaumermarkus.80@gmail.com (M.N.); L.Teplizkaja@web.de (L.T.); 2 Department of Biological Applications and Technology, School of Health Sciences, University of Ioannina, 45110 Ioannina, Greece; 3 Biomedical Research Division, Institute of Molecular Biology and Biotechnology, Foundation for Research and Technology-Hellas (FORTH), 45110 Ioannina, Greece

**Keywords:** anxiety, metabolomics, depression, psychiatry, C57BL/6, DBA/2

## Abstract

Mouse models are widely used to study behavioral phenotypes related to neuropsychiatric disorders. However, different mouse strains vary in their inherent behavioral and molecular characteristics, which needs to be taken into account depending on the nature of the study. Here, we performed a detailed behavioral and molecular comparison of C57BL/6 (B6) and DBA/2 (DBA) mice, two inbred strains commonly used in neuropsychiatric research. We analyzed anxiety-related and depression-like traits, quantified hippocampal and plasma metabolite profiles, and assessed total antioxidant capacity (ΤAC). B6 mice exhibit increased depression-like and decreased anxiety-related behavior compared to DBA mice. Metabolite level differences indicate alterations in amino acid, nucleotide and mitochondrial metabolism that are accompanied by a decreased TAC in B6 compared to DBA mice. Our data reveal multiple behavioral and molecular differences between B6 and DBA mouse strains, which should be considered in the experimental design for phenotype, pharmacological and mechanistic studies relevant for neuropsychiatric disorders.

## 1. Introduction

Mouse models have long been used for a variety of applications in biomedical research. It is well-established that different mouse strains vary in their emotionality profiles and responses to pharmacological treatment [1,2]. B6 and DBA mice are comprehensively characterized, inbred strains, which have been extensively used to study neuropsychiatric phenotypes [3]. Besides being inbred strains, thus ensuring limited heterogeneity, B6 and DBA mice have been consistently shown to differ in their depression-like and anxiety-related behavior [3,4,5]. Divergences in pertinent characteristics such as defensive mechanisms and responses to environmental conditions have been thoroughly investigated [6,7]. B6 and DBA mice also differ in a variety of other aspects, including learning and memory, neuroanatomical correlates, and neurochemical systems [4]. Therefore, these two strains provide a reliable tool to study the molecular correlates of depression and anxiety-related behaviors.

Inherent strain differences both at the behavioral and molecular levels need to be a priori assessed in detail and taken into consideration in experimental design. This is critical for the study of disease-relevant outcomes across laboratories and for ensuring reproducibility. There is only limited information on the underlying molecular differences between these two strains under basal conditions. Existing molecular studies have predominantly focused on alterations in neurotransmitter systems [8,9]. However, brain and plasma metabolite profile differences between basal B6 and DBA mice remain, by and large, unexplored.

Here, we analyzed adult male B6 and DBA mice with a behavioral test battery. We assessed anxiety-related behavior with the dark-light box (DL) and open field (OF) as well as depression-like behavior with the tail suspension test (TST). We then investigated metabolite level changes in hippocampus and plasma using a targeted, mass spectrometry metabolomics platform quantifying up to 300 metabolites [10]. We also assessed oxidative stress by measuring total antioxidant capacity (TAC) in plasma, liver and brain.

## 2. Results

**Figure 1:**
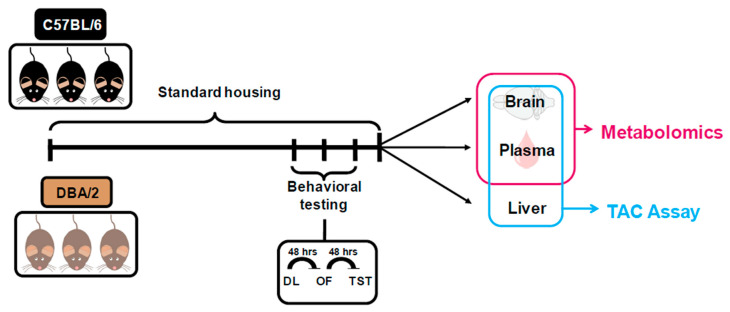
Experimental workflow for comparing B6 and DBA mice. Adult male B6 (*n* = 15) and DBA (*n* = 15) mice were subjected to a behavioral battery (in chronological order DL, OF, TST). Mouse specimens from each strain (15 per group) were collected and used for metabolomics analysis (hippocampus, plasma) and TAC (cortex, plasma, liver). DL: dark-light box; OF: open field; TAC: total antioxidant capacity; TST: tail suspension test.

We compared adult, age-matched male B6 and DBA mice hosted under the same experimental conditions. A behavioral test battery was performed, followed by a metabolomics analysis of hippocampus and plasma [10] and TAC assessment. An overview of the experimental workflow is shown in Figure 1.

### 2.1. Different Behavioral Profiles in B6 and DBA Mice

**Figure 2:**
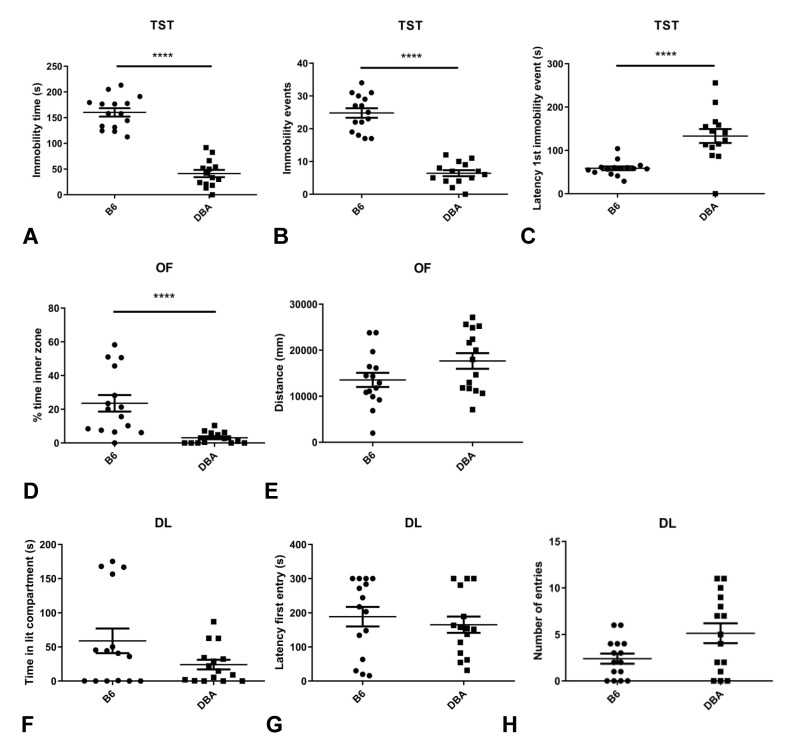
Increased depression-like and decreased anxiety-related behavior in B6 vs. DBA mice. B6 mice exhibit increased depression-like behavior as indicated by increased immobility time (*p* < 0.0001) (**A**), more immobility events (*p* < 0.0001) (**B**) and decreased latency to the first immobility event (*p* < 0.0001) (**C**) compared to DBA mice in TST. B6 mice show decreased anxiety-related behavior as indicated by increased time spent in the inner zone (*p* < 0.0001) (**D**) compared to DBA in OF. Distance travelled in OF did not significantly differ between the two strains (*p* = 0.0890) (**E**). No differences were observed between the two strains in time spent in the lit compartment (*p* = 0.4038) (**F**), latency to the first entry (*p* = 0.6461) (**G**) and number of entries to the lit compartment (*p* = 0.0823) (**H**) in DL (*n* = 15 per group, Mann-Whitney non-parametric test). DL: dark-light box; OF: open field; TST: tail suspension test (**** denotes *p* < 0.0001).

B6 mice show increased depression-like behavior in the TST compared to DBA mice. B6 show an increased immobility time (Figure 2A), an increased number of immobility events (Figure 2B) and sooner reach the first immobility event (Figure 2C) compared to DBA mice. On the other hand, B6 mice show decreased anxiety-related behavior in the OF, as they spend more time in the OF inner zone compared to DBA mice (Figure 2D). Importantly, the distance travelled in the OF is not significantly different between the two strains, indicating that the observed anxiety-related differences are independent of the locomotion of the animals (Figure 2E). No differences between B6 and DBA mice are observed in the DL, including the time spent in the DL lit compartment (Figure 2F), the latency to the first entry in the lit compartment (Figure 2G) and the number of entries to the lit compartment (Figure 2H).

### 2.2. Distinct Metabolomic Profiles in B6 and DBA Mice Hippocampi

**Figure 3:**
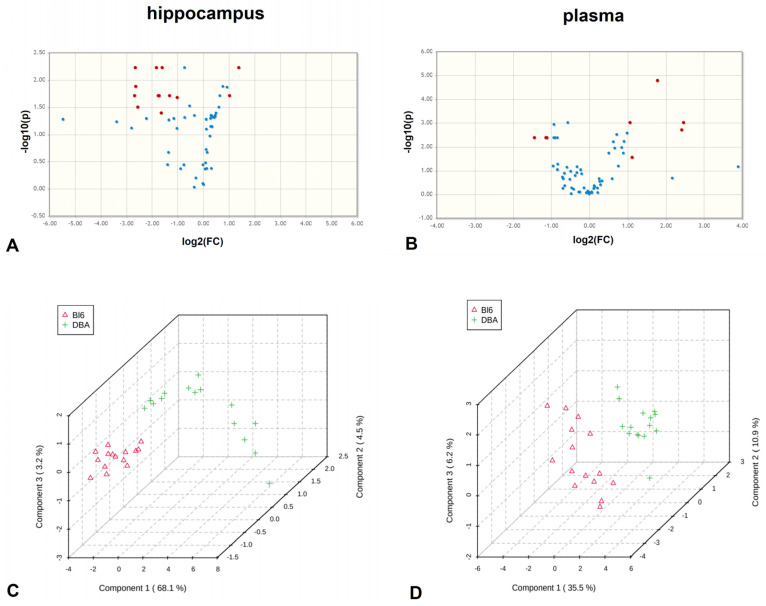
Divergent metabolite profiles for hippocampus and plasma in B6 vs. DBA mice. Volcano plots of significantly altered metabolite levels (false discovery rate (FDR)-corrected *p* < 0.05, unequal group variances) in hippocampus (**A**) and plasma (**B**) of B6 compared to DBA mice. Metabolites with B6/DBA fold change > 2, are highlighted in red and noted with * in Table 1. PLSDA analysis of hippocampus (**C**) and plasma (**D**) as shown by 3D score plots of B6 (Δ) and DBA mice (+). The explained variances are indicated in brackets (hippocampus: 68.1% for component 1, 4.5% for component 2 and 3.2% for component 3; plasma: 35.5% for component 1, 10.9% for component 2, and 3.2% for component 3) (*n* = 15 per group). FC: fold change.

In total, 292 metabolites were measured in B6 and DBA mice hippocampi, of which 276 were considered for metabolomic data analysis (Table S1). Univariate significance analysis of microarrays (SAM) revealed 41 metabolite level differences between B6 and DBA mice (*p* < 0.05, q < 0.01, false discovery rate (FDR) = 0.022) (Table 1). Volcano plots indicated 13 significantly different metabolites in the hippocampus with a fold change >2 (FDR-corrected *p* < 0.05) (Figure 3A, Table 1). These metabolites were also identified in SAM analysis. Multivariate partial least square discriminant analysis (PLSDA) results are shown in Figure 3C.

Of the 41 metabolite level differences identified by SAM, the majority were involved in nucleotide and amino acid metabolism. Changes in nucleotide metabolism included, among others, nucleotide bases (adenine, thymine), other purines besides adenine (hypoxanthine, xanthine), nucleosides (adenosine, inosine) as well as nucleotide components/derivatives (deoxyribose-phosphate, pyrophosphate). Interestingly, we also observed differences of metabolite intermediates that are part of 4 consecutive steps in the major purine catabolism pathway producing uric acid, namely adenosine, inosine, hypoxanthine and xanthine. Adenosine levels were lower in B6, whereas inosine, hypoxanthine and xanthine levels were higher in B6 compared to DBA mice. Changes in amino acid metabolism included amino acids (proline) and amino acid derivatives (betaine, dimethylglycine, pyroglutamic acid, taurine) as well as intermediates of biosynthetic and catabolic amino acid pathways (a-keto-isovalerate, aminoadipic acid, creatinine, S-ribosyl-L-homocysteine).

In addition, metabolite differences were observed for mitochondrial metabolism. We found different levels for 4 out of the 8 intermediates (citrate, isocitrate, fumarate, oxaloacetate) of the tricarboxylic acid cycle (TCA) in mitochondria. Higher citrate and fumarate levels and lower isocitrate and oxaloacetate levels were observed in B6 compared to DBA mice. Importantly, 3 metabolites with significant level differences between B6 and DBA mice (4-aminobutyrate, xanthurenic acid, homocysteic acid) are involved in neurotransmission-related processes.

### 2.3. Distinct Metabolite Profiles in Plasma of B6 and DBA Mice

For plasma, 291 metabolites were measured in B6 and DBA mice, of which 275 were considered for metabolomic data analysis (Table S2). SAM revealed 27 metabolites with significantly different levels in B6 compared to DBA mice (*p* < 0.05, q < 0.01, FDR = 0.020) (Table 1). Eight metabolites with significantly different levels between the two strains were found by volcano plots (fold change > 2, FDR-corrected *p* < 0.05) (Figure 3B, Table 1), and also identified by SAM. PLSDA results are shown in Figure 3D.

The biological process which was predominantly divergent in the plasma of B6 compared to DBA mice is amino acid metabolism. We observed different levels of amino acids (alanine, glutamine, phenylalanine, tryptophan), the tryptophan precursor anthranilate, and modified amino acids/amino acid derivatives (1-methyl-histidine, kynurenic acid, DL-pipecolic acid, N-acetyl-glutamine, *p*-aminobenzoate, taurine). Moreover, differences were found in nucleotide metabolism, including, among others: bases (purine), modified nucleosides (7-methylguanosine), nucleotide components/derivatives (ribose-phosphate, pyrophosphate), intermediates of nucleotide biosynthesis (orotate), as well as uric acid, the main endpoint of purine metabolism. Differences in mitochondria-related processes were also observed and included lower levels of carnitine and acetylcarnitine-DL, which are involved in mitochondrial metabolism of fatty acids, as well as higher levels of the mitochondrial TCA intermediate isocitrate in B6 compared to DBA mice.

### 2.4. Common Metabolite Network Differences in B6 vs. DBA Mice

**Figure 4:**
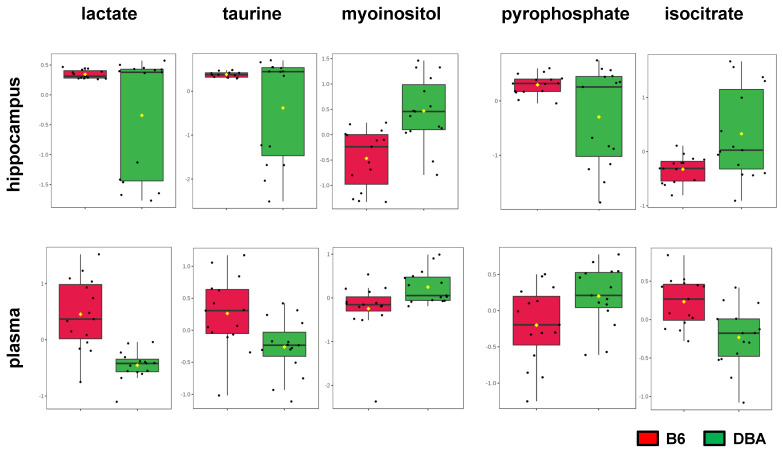
Common metabolite differences in hippocampus and plasma of B6 and DBA mice. Significantly higher levels of lactate and taurine and significantly lower levels of myoinositol in both hippocampus and plasma in B6 compared to DBA mice. Pyrophosphate levels are greater in hippocampus and lower in plasma in B6 compared to DBA mice. Isocitrate levels are lower in hippocampus and higher in plasma in B6 compared to DBA mice. Data are presented as box and whisker plots (*n* = 15 per group).

To identify converging metabolite patterns in the brain and the periphery, we looked for metabolites with significantly altered levels (following SAM) both in hippocampi and plasma between the two strains. We found 5 metabolites with different levels in both specimens (Figure 4, Table 1): lactate, taurine, myoinositol, pyrophosphate and isocitrate. Lactate, taurine and myoinositol levels follow the same direction in both hippocampus and plasma. In particular, lactate and taurine levels are higher, whereas myoinositol levels are lower in B6 compared to DBA mice. Pyrophosphate levels are higher in hippocampus and lower in plasma of B6 compared to DBA mice. The opposite holds true for isocitrate (lower levels in hippocampus and higher levels in plasma of B6 compared to DBA mice).

**Figure 5:**
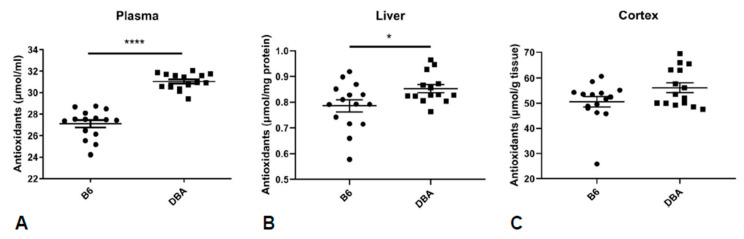
Decreased total antioxidant capacity (TAC) in B6 vs. DBA mice. Decreased TAC in the plasma (*p* < 0.0001, *n* = 15 per group, Mann–Whitney non-parametric test) (**A**), liver (*p* = 0.0263, 15 B6, 14 DBA, *t*-test with Welch’s correction) (**B**) and a trend towards decreased TAC in the cortex (*p* = 0.0574, *n* = 15 per group, *t*-test with Welch’s correction) (**C**) of B6 compared to DBA mice. TAC: total antioxidant capacity (* denotes *p* < 0.05, **** denotes *p* < 0.0001).

Furthermore, as we observed changes in mitochondria-related processes both in brain and plasma and given that oxidative stress is tightly connected to mitochondrial function, we assessed the oxidative stress status of B6 vs. DBA mice. We addressed this in a holistic manner by comparing their TAC, determined as the amount of small molecule and protein antioxidants, in plasma, liver and the brain (Figure 5). Our results show a significantly decreased TAC in B6 vs. DBA mice in plasma and liver, and the same trend in the brain.

## 3. Discussion

We performed a comprehensive behavioral and molecular/biochemical comparison of two common inbred mouse strains, B6 and DBA, which are frequently used in neuropsychiatric research. At the behavioral level, we assessed traits relevant for neuropsychiatric phenotypes, including measurements of depression- and anxiety-related behaviors. We found increased depression-like behavior in TST and decreased anxiety-related behavior in OF in B6 compared to DBA mice. B6 and DBA mice did not differ in their behavior in DL, a finding that is not necessarily surprising, since each test assesses distinct aspects of anxiety-related behavior. Consistent with our findings, B6 male mice also exhibit increased depression-like behavior in the TST and the forced swim test, whereas DBA mice seem to be more sensitive to antidepressant treatment compared to B6 mice [8,9]. B6 adult male mice also show reduced anxiety-related behavior compared to DBA mice in a modified hole board paradigm [3].

Τo explore the underlying molecular differences between B6 and DBA mouse strains, we compared their hippocampal and plasma metabolite profiles. Hippocampus was selected as a brain area of interest due to its role in emotional processing [11], and plasma for its relevance for translational applications. Identified metabolite level differences in both specimens were predominantly involved in amino acid and nucleotide metabolism. Amino acid metabolism is a pathway that has been previously reported to be affected in rodent anxiety models [12,13]. Both amino acid and nucleotide metabolism were also affected in the prefrontal cortex of animal models of depression [14], whereas changes in purine and pyrimidine metabolism were observed in response to antidepressants in mice and patients [15].

In the hippocampus, we identified different levels of TCA cycle intermediates, a fundamental mitochondrial pathway for energy production and cellular respiration, indicating that basal mitochondrial functions responsible for energy homeostasis may differ between B6 and DBA strains. This needs to be considered for experimental procedures investigating neuropsychiatric phenotypes in mice for the following reasons. Firstly, alterations in the TCA cycle have been reported both in patients suffering from psychiatric disorders and in relevant mouse models [16]. Secondly, energy-dependent activities, such as locomotion (or lack thereof), swimming, and struggling are being assessed in behavioral test batteries to determine phenotypic characteristics relevant to psychopathology as well as responses to pharmacological manipulations in mice. Finally, mitochondrial (dys)function has been linked to anxiety [17,18,19,20], stress [21,22] and antidepressant mode of action [23]. Response to chronic stress at the behavioral level was shown to be strain-specific and to be regulated in a mitochondria-dependent manner [24]. We have also previously shown that mitochondrial targeting is a promising pharmacological approach for reducing anxiety levels in vivo [25]. As a result, inherent variations in mitochondrial metabolism across different mouse strains may be a confounding factor in studies aiming to elucidate molecular correlates of mitochondria-driven behavioral changes as well as mitochondria-based therapeutic interventions.

Interestingly, hippocampal differences between B6 and DΒA strains were found for key molecules involved in neurotransmission. These include GABA (4-aminobutyrate), the main inhibitory neurotransmitter, xanthurenic acid, a vesicular glutamate transporter inhibitor, which has been shown to modulate synaptic transmission in the hippocampus [26], and homocysteic acid, a N-methyl-D-aspartic acid (NMDA) receptor agonist. These differences should be taken into account particularly, when using the B6 and DBA strains to study molecular correlates of neural function, as perturbations of the neurotransmission machinery are characteristic for neuropsychiatric disorder pathobiology [27].

Metabolite level differences in plasma included carnitine and acetylcarnitine DL. Besides playing a role in mitochondrial lipid metabolism, carnitine and acetylcarnitine have a protective role against oxidative stress [28]. Altered carnitine and acetylcarnitine DL levels have been reported both in the brain and plasma of high anxiety-related behavior compared to low anxiety-related behavior mice [12], and converging evidence highlights the potential of acetylcarnitine administration in exerting anxiolytic, stress-relieving and antidepressant effects [29,30]. Furthermore, plasma levels of kynurenic acid, a tryptophan derivative, differed between B6 and DBA mice. Plasma kynurenic acid levels have been suggested as a potential predictive and treatment response marker for depression [31,32].

Five metabolites with different levels were identified both in hippocampus and plasma: lactate, taurine, myoinositol, pyrophosphate and isocitrate, which are associated with glucose metabolism and cellular respiration. Taurine has been implicated in antioxidant defense by regulating mitochondrial protein synthesis [33] and given that several other differences were involved in oxidative-stress related processes and mitochondrial metabolism, we went on to investigate TACin the plasma, liver and brain of B6 and DBA mice. Here, we focused on cortex, another brain region related to emotional processing [11]. We found a consistent decrease in TAC in B6 vs. DBA mice in all specimens studied. As oxidative stress-related changes have been reported in peripheral specimens in psychiatric disorders [34], basal oxidative status differences between B6 and DBA mice should be taken into account for molecular and pharmacological investigations.

Our results underline the importance of acknowledging inherent basal molecular differences between mouse strains. Here, by investigating both brain tissue and peripheral specimens using a holistic approach, we highlight the need to identify divergent molecular signatures at a systemic level and expand beyond selected neurotransmitter-related processes. This framework will allow us to better understand pathology relevant to neuropsychiatric phenotypes, as neuropsychiatric disorders are complex multifactorial conditions encompassing perturbations of multiple pathways. In our study, brain metabolome data were assessed in hippocampus and TAC data in the cortex. Additional investigations need to be performed in other brain regions relevant to emotional processing in order to achieve a more comprehensive map of molecular strain differences. Furthermore, more targeted methodologies can be used for metabolites of interest in order to pinpoint whether specific metabolite levels correlate with behavioral parameter changes. Finally, it will be critical to compare the strain-specific molecular changes identified here with data from patients suffering from depression and anxiety disorders in order to detect converging molecular signatures.

## 4. Material and Methods

### 4.1. Animals

The animal experiments were approved by local authorities and conducted according to current regulations for animal experimentation in Germany and the European Union (European Communities Council Directive 2010/63/EU). Three-week-old, male B6 mice (*n* = 15, C57BL/6N, Martinsried, Germany) and DBA mice (*n* = 15, DBA/2, Charles River, Sulzfeld, Germany) were purchased and housed in groups of three for 11 weeks at the animal facility of the *Max Planck Institute of Psychiatry* under standard conditions as previously described [35].

### 4.2. Behavioral Testing and Sample Collection

During the 15th week of age, DL and OF were performed to assess anxiety-related behavior, followed by TST to assess depression-like behavior, as previously described [25]. The time interval among each test was 48 h. Mice were tested in a randomized order, which was maintained for all 3 tests. DL and OF behavioral data were analyzed by ANY-maze (Stoelting Co, Wood Dale, IL, USA). TST was manually scored using Eventlog 1.0 (EMCO Software, Reykjavik, Iceland). Behavioral testing and analysis were performed by the same experienced experimenter. Animals used for this analysis served as controls in a previously published pharmacological experiment [25]. After behavioral testing, hippocampus, cortex and plasma were collected as previously described [36] and stored at −80 °C.

### 4.3. Targeted Metabolomics Sample Preparation and Measurement

Plasma and hippocampal samples were prepared for metabolomics analysis as previously described [12]. Targeted metabolomics measurements were performed at the Mass Spectrometry Core of Beth Israel Deaconess Medical Center, Harvard Medical School (Boston, MA, USA) as previously described [25] using a 550 QTRAP triple quadrupole mass spectrometer (AB/Sciex, Framingham, MA, USA) coupled to a Prominence UFCL HPLC system (Shimadzu, Columbia, MD, USA). The platform used is based on SRM of selected metabolites as previously outlined [10].

### 4.4. Targeted Metabolomics Data Analysis

Metabolite data were analyzed by MetaboAnalyst 4.0 (https://www.metaboanalyst.ca (accessed on 2 February 2021)) [37]. Both for hippocampi and plasma datasets the following analysis workflow was implemented. For metabolites measured both in positive and negative mode, the mode with fewer missing values was selected. When the same number of missing values was observed, then the mode with the higher intensities was included for the analysis. Metabolites with >30% missing values were not included in the analysis. Variables with missing values were excluded and no data filtering was applied. Data were median-normalized, log-transformed and Pareto-scaled.

For volcano plots, metabolite differences > 2 fold with an FDR-corrected *p* < 0.05 (unequal variances) were considered significant. Data were analyzed using a multivariate (PLSDA) and a univariate (SAM) method. For SAM analysis, important features with FDR < 0.05, *p* < 0.05 and q < 0.01 were considered.

### 4.5. Total Antioxidant Capacity

Plasma and cortex from 15 mice per group and liver from 15 B6 and 14 DBA mice were analyzed by a TAC assay kit (K274-100, BioVision, Mountain View, CA, USA) as previously described [18].

### 4.6. Statistical Analysis

Behavioral and TAC assay plasma data were analyzed by the non-parametrical, Mann-Whitney test. TAC assay liver and cortex data were analyzed by *t*-test with Welch’s correction, having passed at least one of the following normality tests (Anderson-Darling, D’Agostino-Pearson, Shapiro-Wilk, Kolmogorov-Smirnov). Data are presented as mean ± SEM. Statistical analysis was performed by GraphPad Prism 8 (GraphPad Software, San Diego, CA, USA).

## 5. Conclusions

Taken together, we show that B6 and DBA mouse strains are characterized by divergent basal brain and plasma metabolite signatures. Small molecules and pathway differences between the two strains have been implicated in depression- and anxiety-related phenotypes and/or are involved in mechanisms of antidepressant or anxiolytic treatments. Therefore, these inherent variations should be considered for the experimental design and choice of mouse strain to ensure a valid result interpretation.

## Figures and Tables

**Table 1:** Significantly different metabolite levels between B6 and DBA mice (*p* < 0.05, q < 0.01) in hippocampus and plasma identified by significance analysis of microarrays (SAM). Metabolite levels different for both specimens are highlighted in bold and italics. Metabolite levels were identified as different by volcano plots are noted with * (*n* = 15 per group).

Hippocampus Metabolites	*p* Value	q Value		Plasma Metabolites	*p* Value	q Value
2-hydroxy-2-methylbutanedioic acid	0.0004	0.0002		1-methyl-histidine	0.0211	0.0078
2-hydroxygluterate	0.0049	0.0014		7-methylguanosine	0.0006	0.0004
2-keto-isovalerate	0.0347	0.0043		acetylcarnitine DL	0.0003	0.0002
2-oxobutanoate*	0.0021	0.0007		alanine	0.0086	0.0039
3-methylphenylacetic acid	0.0256	0.0034		anthranilate*	0.0003	0.0002
4-aminobutyrate	0.0212	0.0030		carnitine	<0.0001	<0.0001
adenine*	0.0032	0.0010		DL-Pipecolic acid	0.0002	0.0002
adenosine*	<0.0001	<0.0001		glutamine*	<0.0001	<0.0001
allantoate	<0.0001	<0.0001		glycerate	0.0009	0.0006
allantoin	0.0179	0.0030		guanidoacetic acid*	<0.0001	<0.0001
aminoadipic acid	0.0425	0.0050		indole	0.0164	0.0066
atrolactic acid	0.0439	0.0051		***isocitrate***	***0.0030***	***0.0017***
betaine	0.0126	0.0025		kynurenic acid	0.0003	0.0002
citrate	0.0465	0.0052		lactate*	***<0.0001***	***<0.0001***
creatinine*	<0.0001	<0.0001		***myo-inositol***	***0.0113***	***0.0047***
deoxyribose-phosphate	0.0244	0.0033		N-acetyl-glutamine	0.0019	0.0011
D-glucarate	0.0212	0.0030		orotate	0.0111	0.0047
D-glyceraldehdye-3-phosphate	0.0007	0.0004		p-aminobenzoate*	0.0003	0.0002
dimethylglycine*	0.0004	0.0002		phenylalanine	0.0003	0.0002
fructose-1,6-bisphosphate*	<0.0001	<0.0001		purine	0.0003	0.0002
fumarate	0.0339	0.0043		***pyrophosphate***	***0.0244***	***0.0087***
glyoxylate*	0.0018	0.0006		ribose-phosphate	0.0066	0.0032
homocysteic acid	0.0212	0.0030		S-adenosyl-L-methionine*	0.0002	0.0002
hypoxanthine	0.0147	0.0028		sn-glycerol-3-phosphate*	<0.0001	<0.0001
inosine	0.0089	0.0022		taurine*	***0.0066***	***0.0032***
isocitrate*	***0.0060***	***0.0015***		tryptophan	0.0039	0.0021
***lactate***	***0.0126***	***0.0025***		uric acid	0.0208	0.0078
myo-inositol*	***<0.0001***	***<0.0001***				
oxaloacetate*	0.0109	0.0025				
pantothenate*	0.0007	0.0004				
phosphorylcholine	0.0121	0.0025				
proline	0.0161	0.0030				
pyroglutamic acid	0.0058	0.0015				
***pyrophosphate***	***0.0181***	***0.0030***				
SBP	0.0018	0.0006				
S-ribosyl-L-homocysteine*	0.0014	0.0006				
***taurine***	***0.0186***	***0.0030***				
thymine*	0.0014	0.0006				
urea	0.0212	0.0030				
xanthine	0.0356	0.0043				
xanthurenic acid	0.0209	0.0030				

## Data Availability

The data presented in this study are available on request from the corresponding author.

## Supplementary Materials

The following are available online at https://www.mdpi.com/2218-1989/11/2/128/s1, Table S1: Raw hippocampal metabolite data considered for metabolomic analysis, Table S2: Raw plasma metabolite data considered for metabolomic analysis.

## Abbreviations

DL	dark-light box
FDR	false discovery rate
NMDA	N-methyl-D-aspartic acid
OF	open field
PLSDA	partial least square discriminant analysis
SAM	significance analysis of microarrays
SRM	selected reaction monitoring
TAC	total antioxidant capacity
TCA	tricarboxylic acid cycle
TST	tail suspension test

## References

[1] Griebel G., Belzung C., Perrault G., Sanger D.J. (2000). Differences in anxiety-related behaviours and in sensitivity to diazepam in inbred and outbred strains of mice. Psychopharmacology.

[2] Sultana R., Ogundele O.M., Lee C.C. (2019). Contrasting characteristic behaviours among common laboratory mouse strains. R. Soc. Open Sci..

[3] Ohl F., Roedel A., Binder E., Holsboer F. (2003). Impact of high and low anxiety on cognitive performance in a modified hole board test in C57BL/6 and DBA/2 mice. Eur. J. Neurosci..

[4] Podhorna J., Brown R.E. (2002). Strain differences in activity and emotionality do not account for differences in learning and memory performance between C57BL/6 and DBA/2 mice. Genes Brain Behav..

[5] O’Leary T.P., Gunn R.K., Brown R.E. (2013). What are we measuring when we test strain differences in anxiety in mice?. Behav. Genet..

[6] Griebel G., Sanger D.J., Perrault G. (1997). Genetic differences in the mouse defense test battery. Aggress Behav..

[7] Võikar V., Polus A., Vasar E., Rauvala H. (2005). Long-term individual housing in C57BL/6J and DBA/ mice: Assessment of behavioral consequences. Genes Brain Behav..

[8] Sugimoto Y., Kajiwara Y., Hirano K., Yamada S., Tagawa N., Kobayashi Y., Hotta Y., Yamada J. (2008). Mouse strain differences in immobility and sensitivity to fluvoxamine and desipramine in the forced swimming test: Analysis of serotonin and noradrenaline transporter binding. Eur. J. Pharmacol..

[9] Popova N.K., Naumenko V.S., Tibeikina M.A., Kulikov A.V. (2009). Serotonin transporter, 5-HT1A receptor, and behavior in DBA/2J mice in comparison with four inbred mouse strains. J. Neurosci. Res..

[10] Yuan M., Breitkopf S.B., Yang X.M., Asara J.M. (2012). A positive/negative ion-switching, targeted mass spectrometry-based metabolomics platform for bodily fluids, cells, and fresh and fixed tissue. Nat. Protoc..

[11] McEwen B.S., Nasca C., Gray J.D. (2016). Stress Effects on Neuronal Structure: Hippocampus, Amygdala, and Prefrontal Cortex. Neuropsychopharmacology.

[12] Filiou M.D., Asara J.M., Nussbaumer M., Teplytska L., Landgraf R., Turck C.W. (2014). Behavioral extremes of trait anxiety in mice are characterized by distinct metabolic profiles. J. Psychiatr. Res..

[13] Humer E., Probst T., Pieh C. (2020). Metabolomics in Psychiatric Disorders: What We Learn from Animal Models. Metabolites.

[14] Tian L., Pu J., Liu Y., Gui S., Zhong X., Song X., Xu S., Zhang H., Wang H., Zhou W. (2020). Metabolomic analysis of animal models of depression. Metab. Brain Dis..

[15] Park D.I., Dournes C., Sillaber I., Uhr M., Asara J.M., Gassen N.C., Rein T., Ising M., Webhofer C., Filiou M.D. (2016). Purine and pyrimidine metabolism: Convergent evidence on chronic antidepressant treatment response in mice and humans. Sci. Rep..

[16] Vlaikou A.M., Nussbaumer M., Komini C., Lambrianidou A., Konidaris C., Trangas T., Filiou M.D. (2020). Exploring the crosstalk of glycolysis and mitochondrial metabolism in psychiatric disorders and brain tumors. Eur. J. Neurosci..

[17] Filiou M.D., Sandi C. (2019). Anxiety and Brain Mitochondria: A Bidirectional Crosstalk. Trends Neurosci..

[18] Filiou M.D., Zhang Y., Teplytska L., Reckow S., Gormanns P., Maccarrone G., Frank E., Kessler M.S., Hambsch B., Nussbaumer M. (2011). Proteomics and metabolomics analysis of a trait anxiety mouse model reveals divergent mitochondrial pathways. Biol. Psychiatry.

[19] Iris F., Filiou M., Turck C.W. (2014). Differential proteomics analyses reveal anxiety-associated molecular and cellular mechanisms in cingulate cortex synapses. AJPN.

[20] Zhang Y., Filiou M.D., Reckow S., Gormanns P., Maccarrone G., Kessler M.S., Frank E., Hambsch B., Holsboer F., Landgraf R. (2011). Proteomic and metabolomic profiling of a trait anxiety mouse model implicate affected pathways. Mol. Cell Proteom..

[21] Lopes S., Teplytska L., Vaz-Silva J., Dioli C., Trindade R., Morais M., Webhofer C., Maccarrone G., Almeida O.F.X., Turck C.W. (2017). Tau Deletion Prevents Stress-Induced Dendritic Atrophy in Prefrontal Cortex: Role of Synaptic Mitochondria. Cereb. Cortex.

[22] Picard M., McEwen B.S., Epel E.S., Sandi C. (2018). An energetic view of stress: Focus on mitochondria. Front. Neuroendocrinol..

[23] Weckmann K., Deery M.J., Howard J.A., Feret R., Asara J.M., Dethloff F., Filiou M.D., Iannace J., Labermaier C., Maccarrone G. (2017). Ketamine’s antidepressant effect is mediated by energy metabolism and antioxidant defense system. Sci. Rep..

[24] Misiewicz Z., Iurato S., Kulesskaya N., Salminen L., Rodrigues L., Maccarrone G., Martins J., Czamara D., Laine M.A., Sokolowska E. (2019). Multi-omics analysis identifies mitochondrial pathways associated with anxiety-related behavior. PLoS Genet..

[25] Nussbaumer M., Asara J.M., Teplytska L., Murphy M.P., Logan A., Turck C.W., Filiou M.D. (2016). Selective Mitochondrial Targeting Exerts Anxiolytic Effects In Vivo. Neuropsychopharmacology.

[26] Neale S.A., Copeland C.S., Uebele V.N., Thomson F.J., Salt T.E. (2013). Modulation of hippocampal synaptic transmission by the kynurenine pathway member xanthurenic acid and other VGLUT inhibitors. Neuropsychopharmacology.

[27] Sarter M., Bruno J.P., Parikh V. (2007). Abnormal neurotransmitter release underlying behavioral and cognitive disorders: Toward concepts of dynamic and function-specific dysregulation. Neuropsychopharmacology.

[28] Maldonado C., Vazquez M., Fagiolino P. (2020). Potential Therapeutic Role of Carnitine and Acetylcarnitine in Neurological Disorders. Curr. Pharm. Des..

[29] Cherix A., Larrieu T., Grosse J., Rodrigues J., McEwen B., Nasca C., Gruetter R., Sandi C. (2020). Metabolic signature in nucleus accumbens for anti-depressant-like effects of acetyl-L-carnitine. eLife.

[30] Pancotto L., Mocelin R., Marcon M., Herrmann A.P., Piato A. (2018). Anxiolytic and anti-stress effects of acute administration of acetyl-L-carnitine in zebrafish. PeerJ.

[31] Erabi H., Okada G., Shibasaki C., Setoyama D., Kang D., Takamura M., Yoshino A., Fuchikami M., Kurata A., Kato T.A. (2020). Kynurenic acid is a potential overlapped biomarker between diagnosis and treatment response for depression from metabolome analysis. Sci. Rep..

[32] Liu H., Ding L., Zhang H., Mellor D., Wu H., Zhao D., Wu C., Lin Z., Yuan J., Peng D. (2018). The Metabolic Factor Kynurenic Acid of Kynurenine Pathway Predicts Major Depressive Disorder. Front. Psychiatry.

[33] Jong C.J., Azuma J. (2012). Schaffer, Mechanism underlying the antioxidant activity of taurine: Prevention of mitochondrial oxidant production. Amino Acids.

[34] Papadopoulou Z., Vlaikou A.M., Theodoridou D., Komini C., Chalkiadaki G., Vafeiadi M., Margetaki K., Trangas T., Turck C.W., Syrrou M. (2019). Unraveling the Serum Metabolomic Profile of Post-partum Depression. Front. Neurosci..

[35] Frank E., Kessler M.S., Filiou M.D., Zhang Y., Maccarrone G., Reckow S., Bunck M., Heumann H., Turck C.W., Landgraf R. (2009). Stable isotope metabolic labeling with a novel ^15^N-enriched bacteria diet for improved proteomic analyses of mouse models for psychopathologies. PLoS ONE.

[36] Filiou M.D., Teplytska L., Otte D.M., Zimmer A., Turck C.W. (2012). Myelination and oxidative stress alterations in the cerebellum of the G72/G30 transgenic schizophrenia mouse model. J. Psychiatr. Res..

[37] Chong J., Soufan O., Li C., Caraus I., Li S., Bourque G., Wishart D.S., Xia J. (2018). MetaboAnalyst 4.0: Towards more transparent and integrative metabolomics analysis. Nucleic Acids Res..

